# Metabolic reprogramming orchestrates microglial fate in cerebral ischemia-reperfusion injury: a spatiotemporal immunometabolic perspective

**DOI:** 10.3389/fimmu.2026.1831494

**Published:** 2026-07-23

**Authors:** Meng Zhang, Huimin Li, Wei Zhang

**Affiliations:** 1 Tongde Hospital of Zhejiang Province Affiliated to Zhejiang Chinese Medical University, Hangzhou, Zhejiang, China; 2 Zhejiang Academy of Traditional Chinese Medicine, Hangzhou, Zhejiang, China

**Keywords:** cerebral ischemia-reperfusion injury, immunometabolism, metabolic reprogramming, microglia, neuroinflammation, redox signaling

## Abstract

Cerebral ischemia-reperfusion injury (CIRI) represents a critical pathological cascade that paradoxically exacerbates neurological damage following revascularization therapy for acute ischemic stroke (AIS). The pathogenesis of CIRI is intricately linked to dysregulated neuroinflammation, with microglia—the resident innate immune cells of the central nervous system—serving as central orchestrators of this response. Emerging evidence indicates that microglia undergo profound metabolic reprogramming encompassing glucose metabolism, the tricarboxylic acid (TCA) cycle, fatty acid metabolism, and NAD^+^ homeostasis, which fundamentally dictates their functional polarization and consequent neuroinflammatory outcomes. Rather than existing as discrete pro-inflammatory versus reparative phenotypes (classically referred to as M1/M2), microglia exhibit a continuum of activation states with distinct metabolic signatures that evolve dynamically across spatiotemporal dimensions following CIRI. Here, we systematically synthesize current knowledge on the core molecular mechanisms underlying microglial metabolic reprogramming, including the ACOD1/itaconate pathway, the glycolysis-OxPhos balance, and NAMPT-mediated NAD^+^ homeostasis. We critically examine the intricate crosstalk between these metabolic pathways and neuroinflammatory signaling cascades, revealing how metabolic checkpoints serve as integrative nodes that decode microenvironmental cues into functional outputs. Building on this mechanistic foundation, we evaluate emerging intervention strategies targeting metabolic reprogramming, stratified by intervention modality and translational readiness, with emphasis on agents in active clinical development. Finally, we identify prevailing challenges—including spatiotemporal heterogeneity, cell-specific targeting requirements, and clinical translation barriers—and outline future directions integrating single-cell omics, systems biology approaches, and advanced delivery systems. This comprehensive analysis aims to provide a refined conceptual framework and highlight promising therapeutic avenues for mitigating CIRI through strategic modulation of microglial immunometabolism.

## Graphical Abstract

**Figure:**
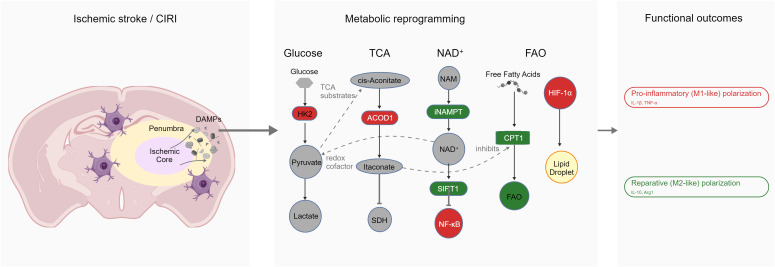
Schematic overview of microglial metabolic reprogramming in cerebral ischemia-reperfusion injury. Following ischemic stroke and subsequent reperfusion, the ischemic core and penumbra release damage-associated molecular patterns (DAMPs), which trigger microglial activation and metabolic reprogramming. Four interconnected metabolic pathways—glucose metabolism, the TCA cycle/itaconate axis, NAD^+^ metabolism, and fatty acid metabolism—orchestrate microglial functional polarization. In glucose metabolism, HK2 drives glycolysis, producing pyruvate and lactate. The TCA cycle converts cis-aconitate to itaconate, which inhibits succinate dehydrogenase (SDH). NAD^+^ metabolism involves iNAMPT-mediated salvage synthesis, with NAD^+^ serving as a substrate for SIRT1, which inhibits NF-κB. Fatty acid metabolism includes CPT1-mediated fatty acid oxidation and HIF-1α-driven lipid droplet accumulation. Crosstalk between pathways is indicated by gray dashed arrows: pyruvate provides carbon skeletons for the TCA cycle; itaconate inhibits CPT1; and NAD^+^ serves as a redox cofactor for glycolysis. These metabolic changes ultimately determine microglial fate, driving either pro-inflammatory (classically M1-like) polarization characterized by IL-1β and TNF-α production, or reparative (classically M2-like) polarization characterized by IL-10 and Arg1 expression. Red elements denote pro-inflammatory signals/phenotypes; blue/green elements denote anti-inflammatory/reparative signals/phenotypes.

## 1 Introduction

Acute ischemic stroke (AIS) remains a leading global cause of mortality and long-term disability, with reperfusion therapies—including intravenous thrombolysis and mechanical thrombectomy—representing the cornerstone of acute management (1). While timely restoration of cerebral blood flow is essential for salvaging ischemic tissue, the ensuing reperfusion phase paradoxically initiates a complex cascade of secondary injuries collectively termed cerebral ischemia-reperfusion injury (CIRI), which substantially undermines neurological recovery and clinical outcomes (2). The pathophysiology of CIRI is multifaceted, involving excitotoxicity, oxidative stress, calcium dysregulation, mitochondrial dysfunction, and a robust neuroinflammatory response (3). Within this complex pathological landscape, microglia-driven neuroinflammation has emerged as both a critical amplifier of tissue damage and a key determinant of functional prognosis (4, 5).

As the primary innate immune sentinels of the central nervous system (CNS), microglia in the healthy brain exist in a highly dynamic surveillant state, continuously monitoring the parenchymal microenvironment through motile processes (6). Following CIRI, the ischemic core and penumbra release a plethora of damage-associated molecular patterns (DAMPs), including HMGB1, ATP, and S100 proteins, which engage pattern recognition receptors on microglia and trigger rapid activation (7). This activation is not merely a functional switch but is underpinned by a profound reorganization of cellular metabolism—termed metabolic reprogramming—that enables microglia to meet the bioenergetic and biosynthetic demands of activation while simultaneously tailoring their functional output (8, 9). The burgeoning field of immunometabolism has revealed that metabolic pathways function not as passive support systems but as active regulators of immune cell fate and function, with specific metabolic checkpoints and metabolites directly influencing inflammatory signaling cascades (10).

Traditional conceptual frameworks have classified activated microglia into pro-inflammatory (M1-like) and reparative (M2-like) phenotypes, with M1-like cells relying on aerobic glycolysis (the Warburg effect) to fuel rapid production of ATP and pro-inflammatory mediators, while M2-like cells utilize oxidative phosphorylation (OxPhos) and fatty acid β-oxidation (FAO) to support sustained energy demands for phagocytosis and tissue repair (9, 11). However, advanced single-cell transcriptomic and metabolomic studies have fundamentally challenged this binary paradigm, revealing that microglia exist along a continuum of activation states with considerable heterogeneity and plasticity (12, 13). Distinct disease-associated microglial subpopulations—including ischemia-associated microglia—have been identified, each characterized by unique metabolic signatures and functional properties that evolve dynamically across the spatiotemporal trajectory of CIRI (13, 14).

Throughout this review, we use the terms “pro-inflammatory” and “reparative” to describe functional poles of the microglial activation continuum, while noting that the classical M1/M2 nomenclature—though conceptually useful historically—represents an oversimplification of the actual *in vivo* heterogeneity.

Herein, we provide a comprehensive synthesis of current knowledge on microglial metabolic reprogramming in CIRI, with three principal objectives: (i) to delineate the dynamic spatiotemporal landscape of microglial activation and the integrated molecular network governing metabolic reprogramming; (ii) to critically examine the core metabolic pathways and their intricate crosstalk, addressing controversial findings and context-dependent effects; and (iii) to evaluate emerging therapeutic strategies targeting metabolic reprogramming, with emphasis on agents in clinical development, while identifying key challenges and future directions for the field (Graphical Abstract).

## 2 The microglial response and neuroinflammatory network in CIRI: A spatiotemporal perspective

**Figure 1:**
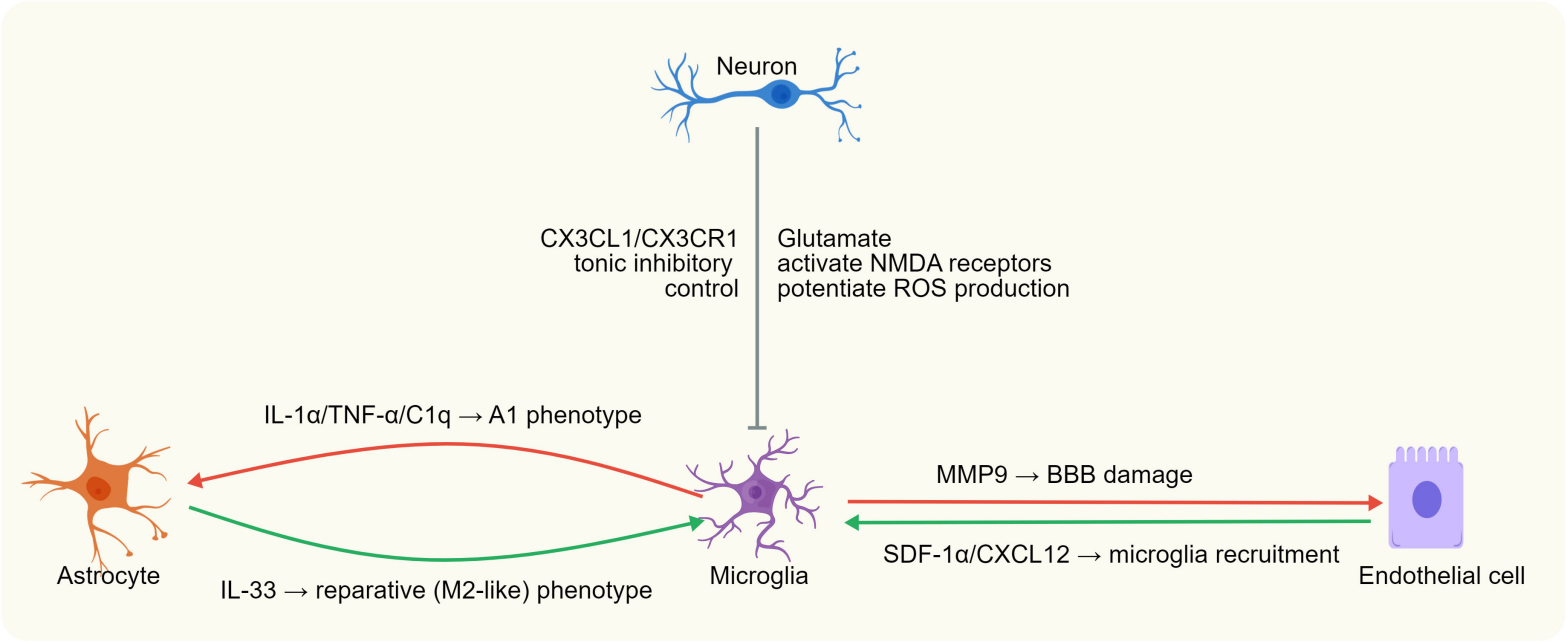
Microglial crosstalk within the neurovascular unit following CIRI. Schematic representation of bidirectional communication between microglia and neighboring cells in the neurovascular unit after cerebral ischemia-reperfusion injury. Neurons exert tonic inhibitory control via the CX3CL1/CX3CR1 axis, while releasing glutamate that activates microglial NMDA receptors and potentiates ROS production. Activated microglia secrete IL-1α, TNF-α, and C1q, driving astrocytes toward a detrimental A1 neurotoxic phenotype. Conversely, astrocytes produce IL-33 to promote microglial Reparative (classically M2-like) polarization. Endothelial cells recruit microglia via SDF-1α/CXCL12, while microglia-derived MMP9 contributes to blood-brain barrier (BBB) disruption. CX3CL1, fractalkine; CX3CR1, fractalkine receptor; IL, interleukin; MMP9, matrix metalloproteinase-9; ROS, reactive oxygen species; SDF-1α, stromal cell-derived factor 1α; TNF-α, tumor necrosis factor-α.

Microglial activation following CIRI is a highly dynamic and context-dependent process shaped by complex microenvironmental cues and bidirectional intercellular communication within the neurovascular unit (**Figure 1**).

### 2.1 Phenotypic heterogeneity and dynamic polarization: beyond the M1/M2 paradigm

While the M1/M2 classification has been conceptually useful, accumulating evidence from single-cell technologies has fundamentally challenged this binary paradigm, revealing a spectrum of activation states that cannot be captured by discrete categories (13, 15). Advanced single-cell transcriptomic studies have unveiled a continuum of activation states with considerable heterogeneity, reflecting a spectrum of functional responses rather than discrete phenotypic categories (12, 16).

**Figure 2:**
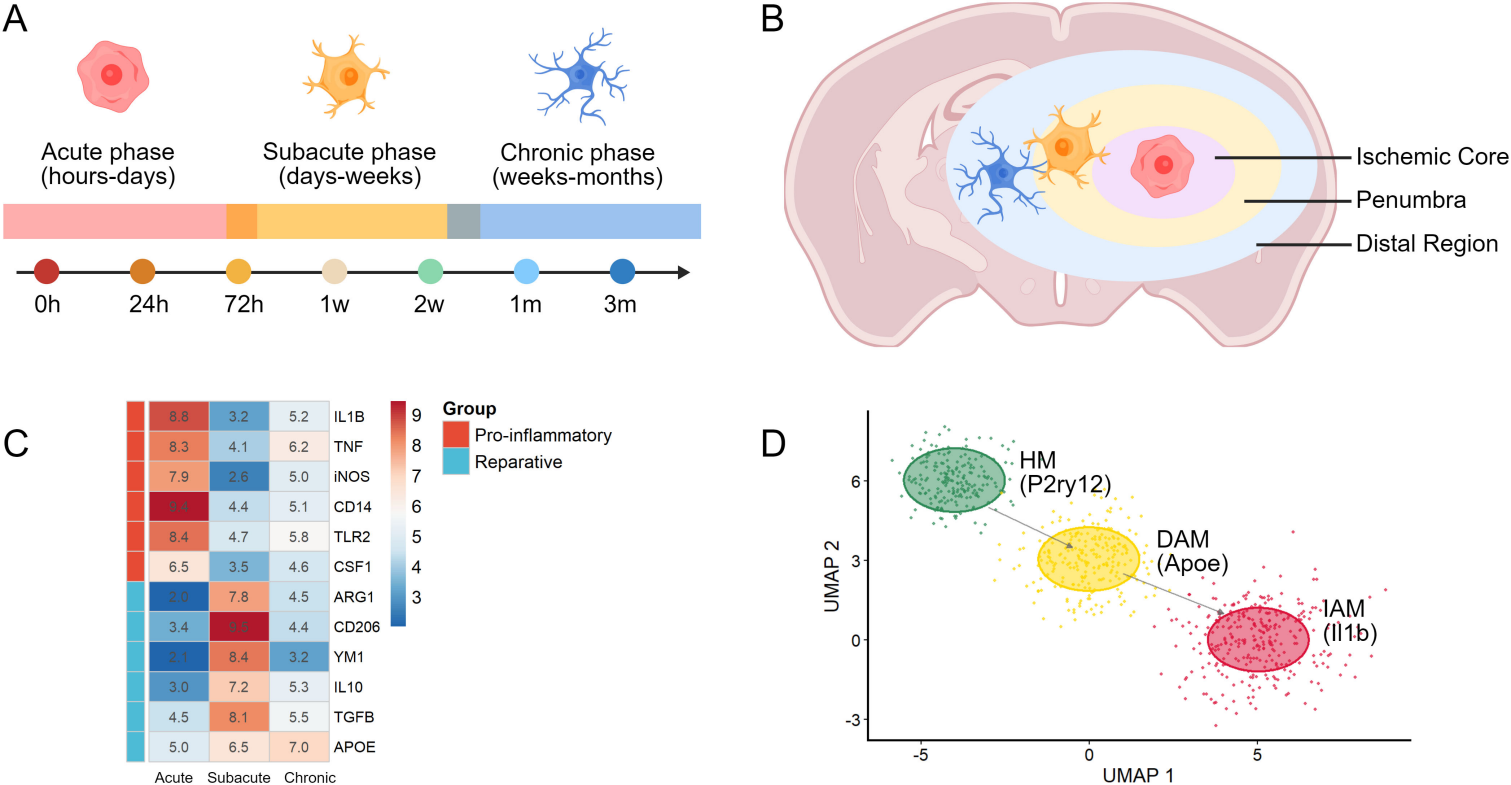
Spatiotemporal dynamics of microglial activation states following CIRI. Following cerebral ischemia-reperfusion injury, microglia exhibit dynamic phenotypic transitions across three temporally overlapping phases. **(A)** Schematic timeline of microglial activation phases: acute (hours to days), subacute (days to weeks), and chronic (weeks to months). **(B)** Representative morphological changes in microglia from ramified (homeostatic) to amoeboid (activated) states in different brain regions relative to the ischemic core. **(C)** Relative expression of pro-inflammatory and reparative markers in microglia during acute, subacute, and chronic phases [based on literature trends (13, 14, 17)]. Blue: low expression; red: high expression. Data are schematic. **(D)** Schematic UMAP visualization of the continuum of microglial activation states in cerebral ischemia-reperfusion injury, including homeostatic microglia (HM), disease-associated microglia (DAM), and ischemia-associated microglia (IAM). The plot illustrates the dynamic transition from homeostatic to pathological activation states, adapted from references (13, 17). (Conceptual schematic based on literature trends; see **Figure 4** for empirical single-cell data). Arg1, arginase 1; CD206, mannose receptor; IL-1β, interleukin-1β; iNOS, inducible nitric oxide synthase; TGF-β, transforming growth factor-β; TNF-α, tumor necrosis factor-α.

Following CIRI, microglia undergo dynamic phenotypic transitions across three temporally overlapping phases (**Figure 2**). In the acute phase (hours to days post-reperfusion), DAMPs engage pattern recognition receptors (TLR2, TLR4) and inflammasome pathways, driving a predominant pro-inflammatory state characterized by nuclear factor kappa-light-chain-enhancer of activated B cells (NF-κB) and mitogen-activated protein kinase (MAPK) activation. Single-cell RNA sequencing has identified distinct ischemia-associated microglial subclusters with upregulated expression of *Cd14*, *Csf1*, *Tlr2*, and *Il1b*, which correlate with infarct volume and neurological deficits (14, 17). During the subacute phase (days to weeks), the microenvironment shifts toward repair, with signals such as interleukin (IL)-4, IL-10, and transforming growth factor-β (TGF-β) promoting microglial transition toward reparative phenotypes associated with tissue remodeling. These cells upregulate markers including Arg1, CD206, and Ym1/2, enhancing phagocytic clearance of cellular debris (5, 18). In the chronic phase (weeks to months), failure of adequate resolution can lead to maladaptive microglial states characterized by persistent low-grade inflammation and impaired phagocytosis, contributing to secondary neurodegeneration (19). Importantly, recent evidence has identified sex-dependent differences in microglial responses. While physiological studies reveal baseline sex differences under normal conditions (20), post-stroke analyses show that females exhibit increased microglial ramification at chronic timepoints and enhanced IL-6 release following IFN-γ stimulation—changes not observed in males (21).

### 2.2 Microglial crosstalk within the neurovascular unit

Neurons exert tonic inhibitory control on microglia via the fractalkine (CX3CL1)/CX3CR1 axis, restraining excessive pro-inflammatory polarization under homeostatic conditions (22). Following ischemic injury, damaged neurons release CX3CL1 and excess glutamate, which can activate microglial NMDA receptors and potentiate reactive oxygen species (ROS) production (5, 19). Astrocytes engage in bidirectional crosstalk with microglia—activated microglia release IL-1α, tumor necrosis factor-α (TNF-α), and complement component 1q (C1q), driving astrocytes toward a detrimental A1 neurotoxic phenotype (23) while astrocytes can produce IL-33 to promote microglial reparative polarization through ST2-dependent signaling (24). Endothelial cells of the blood-brain barrier (BBB) both respond to and influence microglial activity. Microglia-derived matrix metalloproteinase-9 (MMP9) contributes to BBB breakdown (25), while endothelial cells release stromal cell-derived factor 1α (SDF-1α)/CXCL12 to recruit microglia to perivascular sites (5).

Beyond receptor-mediated signaling, metabolic crosstalk between cell types plays an equally important role. The lactate shuttle exemplifies such intercellular metabolic communication: activated microglia produce and release lactate via aerobic glycolysis, which can be taken up by adjacent neurons through monocarboxylate transporters (MCTs) as an alternative energy substrate (26). However, excessive lactate accumulation during acute inflammation may contribute to acidosis and neuronal injury, highlighting context-dependent effects. Astrocytes and microglia also compete for glucose and glutamine; during CIRI, reactive astrocytes upregulate glycolysis and release lactate that may either fuel microglial activation or support neuronal survival depending on the temporal phase. Furthermore, microglial-derived itaconate can be exported and taken up by astrocytes, where it activates Nrf2-mediated antioxidant responses (27), representing a protective metabolic signaling axis. Endothelial cells of the blood-brain barrier (BBB) both respond to and influence microglial activity; metabolic changes in endothelial cells—including glycolysis upregulation and mitochondrial dysfunction—affect nutrient and oxygen delivery to the parenchyma, indirectly shaping microglial metabolism. Thus, metabolic reprogramming in CIRI is not a cell-autonomous phenomenon but rather an integrated network response involving coordinated metabolic adaptations across the entire neurovascular unit.

## 3 Core mechanisms of metabolic reprogramming in microglia during CIRI: an integrated network perspective

### 3.1 Glucose metabolism: the glycolysis-OxPhos axis

**Figure 3:**
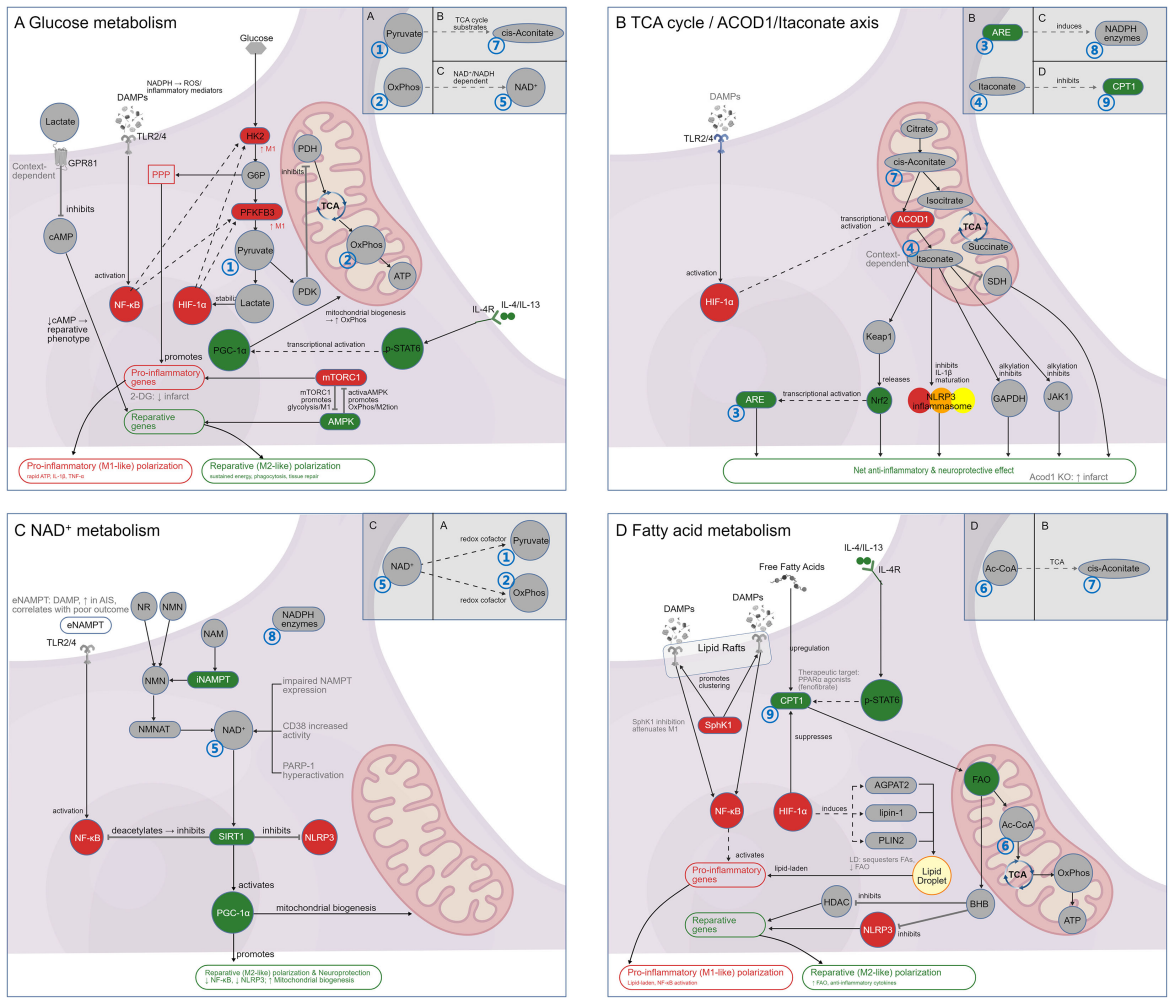
Core metabolic pathways governing microglial fate in cerebral ischemia-reperfusion injury (CIRI). The image is organized into four interconnected subfigures **(A–D)** depicting the major metabolic pathways that orchestrate microglial functional polarization. Red elements denote pro-inflammatory signals/phenotypes; blue/green elements denote anti-inflammatory/reparative signals/phenotypes; blunt-ended lines indicate inhibitory regulation; gray dashed arrows indicate crosstalk between pathways (detailed below). **(A)** Glucose metabolism: Pro-inflammatory (classically M1-like) microglia upregulate hexokinase 2 (HK2) and 6-phosphofructo-2-kinase/fructose-2,6-biphosphatase 3 (PFKFB3) to drive aerobic glycolysis (Warburg effect), supporting rapid ATP production and biosynthesis of inflammatory mediators. Glycolytic flux is diverted into the pentose phosphate pathway (PPP), generating NADPH for reactive oxygen species (ROS) production. Accumulated lactate stabilizes hypoxia-inducible factor-1α (HIF-1α), which further enhances glycolytic gene expression. In parallel, lactate exerts context-dependent biphasic effects: during the acute phase, intracellular lactate promotes HIF-1α stabilization and pro-inflammatory responses; in the subacute phase, extracellular lactate activates G protein-coupled receptor 81 (GPR81), reducing cyclic AMP (cAMP) and promoting reparative gene expression. Reparative (classically M2-like) microglia rely on mitochondrial oxidative phosphorylation (OxPhos), fueled by pyruvate dehydrogenase (PDH), which is inhibited by pyruvate dehydrogenase kinase (PDK) under inflammatory conditions. The IL-4/IL-13 axis activates STAT6, which transcriptionally upregulates PGC-1α, leading to mitochondrial biogenesis. The energy sensors AMPK and mTORC1 mutually antagonize each other, with AMPK promoting OxPhos and reparative polarization, while mTORC1 favors glycolysis and pro-inflammatory polarization. Pharmacological glycolysis inhibition with 2-deoxy-D-glucose (2-DG) reduces infarct volume and apoptosis in rat CIRI models (annotated). **(B)** TCA cycle reprogramming and the ACOD1/itaconate axis: Damage-associated molecular patterns (DAMPs) engage Toll-like receptor 2/4 (TLR2/4), activating NF-κB, which transcriptionally upregulates aconitate decarboxylase 1 (ACOD1). ACOD1 converts cis-aconitate to itaconate within mitochondria. Itaconate exerts pleiotropic anti-inflammatory effects through four mechanisms: (1) competitive inhibition of succinate dehydrogenase (SDH), reducing succinate-driven ROS; (2) alkylation of Kelch-like ECH-associated protein 1 (Keap1), leading to nuclear factor erythroid 2-related factor 2 (Nrf2) release and transcriptional activation of antioxidant response elements (ARE); (3) alkylation-mediated inhibition of glyceraldehyde-3-phosphate dehydrogenase (GAPDH) and Janus kinase 1 (JAK1); and (4) suppression of the NLR family pyrin domain containing 3 (NLRP3) inflammasome, thereby inhibiting IL-1β maturation. These actions collectively confer a net anti-inflammatory and neuroprotective effect. Context-dependent considerations apply: very high itaconate concentrations may exacerbate mitochondrial dysfunction via excessive SDH inhibition, and sustained itaconate production might limit metabolic flexibility. Genetic ablation of *Acod1* significantly increases infarct volume after transient middle cerebral artery occlusion (tMCAO), underscoring the protective role of endogenous itaconate. **(C)** NAD^+^ metabolism: CIRI induces NAD^+^ depletion through three mechanisms: PARP-1 hyperactivation, increased CD38 activity, and impaired nicotinamide phosphoribosyltransferase (NAMPT) expression. The salvage pathway maintains intracellular NAD^+^ pools via NAMPT (iNAMPT), which converts nicotinamide (NAM) to nicotinamide mononucleotide (NMN), followed by NMNAT-mediated conversion to NAD^+^. Intracellular iNAMPT supports sirtuin 1 (SIRT1) activity, which deacetylates and inhibits NF-κB p65, suppresses NLRP3 inflammasome activation, and deacetylates and activates PGC-1α, promoting mitochondrial biogenesis and reparative polarization. In contrast, extracellular NAMPT (eNAMPT/visfatin) acts as a damage-associated molecular pattern (DAMP), engaging TLR4 to exacerbate inflammation (elevated in acute ischemic stroke patients and correlated with poor outcomes). NAD^+^ availability critically influences both glycolysis and oxidative phosphorylation, linking NAD^+^ metabolism to the core energy-producing pathways in microglia (**Figure 3**, crosstalk annotation ②⑤). Therapeutic supplementation with NAD^+^ precursors (NMN, nicotinamide riboside, NR) restores NAD^+^ pools and promotes neuroprotection. **(D)** Fatty acid metabolism: Reparative microglia upregulate carnitine palmitoyltransferase 1 (CPT1) via IL-4/STAT6 signaling, enhancing fatty acid β-oxidation (FAO) within mitochondria. FAO generates acetyl-CoA, fueling the TCA cycle and OxPhos, and produces β-hydroxybutyrate (BHB), which inhibits HDAC and NLRP3, promoting anti-inflammatory gene expression. Conversely, pro-inflammatory microglia accumulate lipid droplets (LDs) driven by HIF-1α, which transcriptionally induces AGPAT2, lipin-1, and PLIN2, leading to LD formation and a “lipid-laden” phenotype. HIF-1α also suppresses FAO by inhibiting CPT1. Lipid rafts serve as platforms for TLR4 clustering, facilitated by sphingosine kinase 1 (SphK1), which enhances NF-κB activation and pro-inflammatory gene expression. PPARα agonists (e.g., fenofibrate) upregulate CPT1 and promote FAO, representing a therapeutic strategy. Crosstalk between pathways: Numbered blue indicators denote metabolic and signaling interactions between subfigures. Each subfigure includes a panel in the upper right corner listing its crosstalk connections. ① Pyruvate from glycolysis **(A)** provides carbon skeletons for the TCA cycle in B (cis-aconitate). ② OxPhos activity **(A)** is NAD^+^-dependent, linking to NAD^+^ metabolism in **(C)** ③ ARE-driven antioxidant program **(B)** induces NADPH-generating enzymes in **(C)** ④ Itaconate **(B)** inhibits CPT1-mediated fatty acid oxidation in **(D)** ⑤ NAD^+^ **(C)** serves as a redox cofactor for both glycolysis (pyruvate) and OxPhos in **(A)** ⑥ Acetyl-CoA from FAO **(D)** enters the TCA cycle in B (cis-aconitate). Color coding and symbols: • Red: pro-inflammatory molecules/phenotypes. • Blue/green: anti-inflammatory/reparative molecules/phenotypes. • Gray: metabolic intermediates, enzymes, and annotations. • Black solid arrows: direct metabolic flux or activation. • Black dashed arrows: transcriptional regulation. • Gray blunt-ended lines: inhibition. • Gray dashed arrows: crosstalk between subfigures. • Numbered blue circles: specific crosstalk interactions (see above). Full molecular abbreviations are provided in the main text.

Under basal conditions, microglia primarily generate ATP through oxidative phosphorylation (OxPhos) (9). CIRI-induced hypoxia and inflammatory signaling trigger a rapid metabolic shift intrinsically linked to phenotypic determination (8, 28)(**Figure 3A**).

Aerobic Glycolysis in Pro-inflammatory polarization: Pro-inflammatory (classically M1-like) microglia upregulate glycolytic enzymes including hexokinase 2 (HK2) and 6-phosphofructo-2-kinase/fructose-2,6-biphosphatase 3 (PFKFB3) following Toll-like receptor (TLR) engagement (29). This metabolic configuration provides rapid ATP generation, diverts glucose into the pentose phosphate pathway for NADPH production, and accumulates glycolytic intermediates for biosynthesis of inflammatory mediators (30). Accumulated lactate contributes to the stabilization of hypoxia-inducible factor-1α (HIF-1α), promoting glycolytic gene expression and inflammatory cytokine production (31). However, lactate also exerts context-dependent anti-inflammatory effects: in the subacute phase, lactate can activate G protein-coupled receptor 81 (GPR81) on microglia, suppressing cyclic AMP (cAMP) and promoting a reparative phenotype (32), and can serve as an alternative energy substrate for neurons via monocarboxylate transporters (MCTs) (26).

Oxidative Phosphorylation in Reparative Polarization: Transition to reparative (classically M2-like) phenotypes is associated with restoration of mitochondrial OxPhos. IL-4/IL-13 activate signal transducer and activator of transcription 6 (STAT6) signaling, promoting mitochondrial biogenesis through peroxisome proliferator-activated receptor gamma coactivator 1α (PGC-1α) (33). AMP-activated protein kinase (AMPK) activation promotes OxPhos and reparative polarization, while mechanistic target of rapamycin complex 1 (mTORC1) signaling enhances glycolysis and pro-inflammatory function (34).

Pathway Crosstalk: Glycolytic flux influences the TCA cycle through pyruvate availability; under inflammatory conditions, pyruvate dehydrogenase is inhibited by pyruvate dehydrogenase kinase (PDK), limiting entry of glucose-derived carbons into the TCA cycle (35). FAO generates acetyl-CoA supporting OxPhos in reparative cells (36), and NAD^+^ availability critically influences both glycolysis and OxPhos (37).

### 3.2 Reprogramming of the TCA cycle: the ACOD1/itaconate axis

The induction of aconitate decarboxylase 1 (*Acod1*; protein: ACOD1, also known as immune-responsive gene 1, IRG1) under inflammatory conditions represents a striking example of metabolic enzymes functioning as immune regulators (38, 39).

The ACOD1/Itaconate Pathway: Following TLR stimulation and NF-κB activation, ACOD1 catalyzes the decarboxylation of cis-aconitate to itaconate (39). Itaconate exerts net anti-inflammatory effects through multiple mechanisms: (i) competitive inhibition of succinate dehydrogenase (SDH); (ii) potent activation of nuclear factor erythroid 2-related factor 2 (Nrf2) via Kelch-like ECH-associated protein 1 (Keap1) alkylation; (iii) direct alkylation of glyceraldehyde-3-phosphate dehydrogenase (GAPDH) (40) and Janus kinase 1 (JAK1); and (iv) inhibition of the NLR family pyrin domain containing 3 (NLRP3) inflammasome (41, 42) (**Figure 3B**). Global *Acod1* knockout significantly increases lesion volumes following transient middle cerebral artery occlusion (tMCAO), indicating that endogenous itaconate plays a protective role during CIRI (43, 44).

Controversies and Context-Dependent Effects: At very high concentrations during acute inflammatory bursts, itaconate-mediated SDH inhibition can exacerbate mitochondrial dysfunction (45), and sustained itaconate production may limit metabolic flexibility for subsequent adaptive responses (46). These findings highlight the importance of optimizing dose and timing for therapeutic applications.

Pathway Crosstalk: Itaconate production couples glycolytic flux to TCA cycle reprogramming, and itaconate itself regulates fatty acid metabolism by inhibiting carnitine palmitoyltransferase 1 (CPT1) activity (47). Itaconate-mediated Nrf2 activation induces antioxidant enzymes and NADPH-generating enzymes (48).

### 3.3 Fatty acid metabolism: β-oxidation, lipid droplets, and membrane dynamics

Fatty Acid β-Oxidation (FAO) and Reparative Polarization: Enhanced FAO is a metabolic signature of reparative microglia (**Figure 3D**), with CPT1 upregulated in response to IL-4/STAT6 signaling (49, 50). FAO generates β-hydroxybutyrate (BHB), which inhibits NLRP3 inflammasome activation and functions as an endogenous histone deacetylase (HDAC) inhibitor, thereby suppressing inflammatory gene expression (51–53). Consistent with this framework, a recent perspective proposes that metabolic reprogramming in ischemic stroke (MRIS) serves as a unifying pathophysiological driver linking acute glycolytic crisis to lipid peroxidation cascades (1).

Lipid Droplets and Metabolic Inflammation: HIF-1α orchestrates lipid droplet (LD) accumulation in microglia following CIRI by inducing triacylglycerol synthesis enzymes (AGPAT2, lipin-1), activating PLIN2 transcription, and suppressing FAO (54, 55). These lipid-laden microglia adopt a pro-inflammatory phenotype contributing to post-ischemic inflammation.

Lipid Rafts in Pro-inflammatory Signaling: Lipid rafts serve as organizing platforms for TLR4 clustering (56). Ischemia-induced oxidative stress alters raft composition, facilitating TLR4 dimerization and NF-κB activation (57). Sphingosine kinase 1 (SphK1) modulates raft organization, and its inhibition attenuates microglial pro-inflammatory activation (58, 59).

Pathway Crosstalk: FAO generates acetyl-CoA entering the TCA cycle; LD formation sequesters fatty acids, reducing FAO substrate availability; cholesterol metabolism influences both raft organization and inflammatory gene expression (60, 61).

### 3.4 NAD^+^ metabolism: a central integrator

NAD^+^ serves as an essential redox cofactor and substrate for sirtuins, poly(ADP-ribose) polymerases (PARPs), and CD38 (37, 62). The salvage pathway enzyme nicotinamide phosphoribosyltransferase (NAMPT) is the primary NAD^+^ source in microglia (63). CIRI induces NAD^+^ depletion through PARP-1 hyperactivation, increased CD38 activity, and impaired NAMPT expression (62, 64) (**Figure 3C**).

Compartment-Specific Functions of NAMPT: Intracellular NAMPT (iNAMPT) maintains NAD^+^ pools, supporting sirtuin 1 (SIRT1)-mediated deacetylation and inhibition of NF-κB p65, suppression of NLRP3, and promotion of PGC-1α-mediated mitochondrial biogenesis (65, 66). Extracellular NAMPT (eNAMPT/visfatin) functions as a DAMP, engaging TLR4 to exacerbate inflammation (67). Elevated plasma eNAMPT in AIS patients correlates with inflammatory markers and poor outcomes (68).

## 4 Therapeutic strategies targeting microglial metabolic reprogramming in CIRI

### 4.1 Targeting metabolic enzymes

Glycolysis Inhibitors: 2-Deoxy-D-glucose (2-DG), an HK2 inhibitor, reduces infarct volume and attenuates microglial pro-inflammatory cytokine production in experimental stroke models (29, 69). In a rat permanent MCAO model, prophylactic administration of 2-DG (100 mg/kg i.p. daily for 7 days prior to ischemia) significantly reduced TUNEL-positive cells and downregulated cleaved-caspase-9 and -3 expression (70), suggesting that glycolysis inhibition may confer preconditioning-like neuroprotection. However, clinical translation of 2-DG is limited by its non-specificity and potential toxicity with prolonged administration, as glycolysis is essential for all cell types (27). Shikonin, a phosphofructokinase 1 (PFK1) inhibitor, shows neuroprotective effects with similar specificity concerns (70).

Itaconate Derivatives: 4-Octyl itaconate (4-OI), a cell-permeable itaconate derivative (which bypasses ACOD1 rather than activating it), consistently reduces infarct volume and promotes microglial reparative polarization in rodent tMCAO models (27, 43). 4-OI activates Nrf2, inhibits NF-κB (27) and NLRP3 inflammasome (71), and is amenable to chemical optimization for improved pharmacokinetics and brain penetration (72). A recent study in neonatal hypoxic-ischemic encephalopathy demonstrated that 4-OI treatment, primarily via astrocytic Nrf2 pathway activation, reduced neuronal death and relieved cognitive dysfunction, with concomitant inhibition of microglial activation (27).

PPAR Agonists: Fenofibrate (peroxisome proliferator-activated receptor α, PPARα agonist), approved for dyslipidemia, upregulates CPT1, enhances FAO, and promotes reparative polarization in experimental stroke (27, 73). A recent study confirmed clofibrate’s anti-neuroinflammatory effects via NF-κB inhibition in ischemic stroke (74). Pioglitazone (PPARγ agonist) similarly promotes reparative polarization (75). These agents offer established human safety profiles, positioning them for drug repurposing. Notably, fenofibrate is under clinical investigation for ischemia-reperfusion injury in other organs, exemplified by a Phase II trial in liver transplantation for preventing ischemic cholangiopathy (NCT05514119), underscoring its translational potential for CIRI.

Targeting Lipid Raft-Associated Proteins: SphK1 inhibitors attenuate TLR4 clustering and Pro-inflammatory polarization (59). PHLDA1 (pleckstrin homology-like domain family A member 1) blockade alleviates CIRI by modulating microglial polarization and NLRP3 activation (76).

### 4.2 Modulating immunometabolic checkpoints

VISTA (V-domain Ig suppressor of T cell activation): VISTA is a negative immune checkpoint on microglia that upregulates ACOD1 expression and itaconate production while suppressing glycolytic flux (77, 78). VISTA agonism reduces infarct volume and improves neurological outcomes in preclinical stroke models (Sun, Y. et al., 2025). Mechanistically, studies in sepsis-associated encephalopathy have revealed that VISTA signaling promotes TRIM28-mediated HK2 ubiquitination and degradation (29), a pathway that may similarly operate in cerebral ischemia.

PD-1/PD-L1 Pathway: Programmed death-ligand 1 (PD-L1) on microglia interacts with programmed cell death protein 1 (PD-1) on infiltrating T cells, delivering inhibitory signals that limit excessive inflammation (79, 80). This axis remains incompletely characterized in CIRI.

### 4.3 Supplementation of key immunomodulatory metabolites

NAD^+^ Precursors: Nicotinamide mononucleotide (NMN) and nicotinamide riboside (NR) supplementation replenishes NAD^+^ pools, enhances SIRT1 activity, and promotes microglial reparative polarization in preclinical models (81, 82). A recent Phase I safety trial (jRCTs041200034) demonstrated that oral NMN administration (250 mg/day for 12 weeks) in healthy subjects is safe and effectively increases blood NAD^+^ levels, supporting its clinical translatability (83).

Itaconate Derivatives: 4-OI and other itaconate-based prodrugs with improved drug-like properties are under preclinical development (72).

Ketone Bodies: BHB administration or ketogenic diet induction shows neuroprotective effects in experimental stroke, suppressing NLRP3 inflammasome and HDAC activity while providing alternative energy substrate (52, 53, 84, 85).

### 4.4 Multi-target natural products

Salidroside, the primary active compound from *Rhodiola rosea*, activates phosphoinositide 3-kinase (PI3K)/Akt/Nrf2 signaling, upregulates ACOD1, inhibits HK2, and promotes microglial reparative polarization (86, 87). While curcumin (88) and celastrol (89, 90) show preclinical efficacy, their clinical translation faces bioavailability challenges (**Table 1**).

**Table 1:** Core metabolic pathways, therapeutic targets, and clinical development status in CIRI.

Metabolic pathway	Key target (s)	Representative agent(s)	Mechanism of action	Preclinical efficacy	Clinical translation stage	Advantages	Limitations	Optimal therapeutic window
Glucose metabolism	HK2, PFKFB3	2-DG (HK2 inhibitor), Shikonin (PFK1 inhibitor)	Inhibit aerobic glycolysis, reduce pro-inflammatory activation	Reduced infarct volume, decreased IL-1β (29, 69, 70)	Preclinical (2-DG); toxicity concerns (27)	Well-characterized mechanism	Non-specific, systemic toxicity risk	Acute (0-72h)
TCA cycle/Itaconate pathway	SDH, Keap1/Nrf2, GAPDH, JAK1	4-Octyl itaconate (4-OI)	Bypasses ACOD1; alkylates Keap1 (activating Nrf2), inhibits SDH, GAPDH, and JAK1, suppresses NLRP3 inflammasome	Reduced infarct size, improved neurological scores, reparative polarization (43, 72)	Preclinical (optimization ongoing) (72)	Pleiotropic anti-inflammatory effects	Pharmacokinetic optimization needed	Acute (0-72h)
Fatty acid metabolism	PPARα, CPT1	Fenofibrate, Clofibrate (PPARα agonists)	Upregulate CPT1, enhance FAO, promote reparative polarization	Improved functional outcomes, reduced neuroinflammation (27, 73, 91)	Fenofibrate: Approved (dyslipidemia); Phase II for hepatic IR injury (NCT05514119) (73). Clofibrate: Preclinical (tMCAO models) (74)	Established safety, oral bioavailability	Tissue non-specific	Subacute (3-14d)
Fatty acid metabolism	PPARγ	Pioglitazone	Enhance fatty acid uptake/storage, promote reparative polarization	Reduced infarct volume, anti-inflammatory (88)	Approved (diabetes)	Established safety	Metabolic side effects	Subacute (3-14d)
NAD^+^ metabolism	NAMPT, SIRT1	NMN, NR	Replenish NAD^+^ pools, activate SIRT1	Decreased oxidative stress, improved mitochondrial function, reparative polarization (81, 82)	Phase II (aging: jRCTs041200034) (83)	Targets multiple pathways simultaneously	Optimal dosing/timing under investigation	Subacute to chronic
Lipid raft signaling	SphK1, PHLDA1	SphK1 inhibitors, PHLDA1 blockers	Disrupt TLR4 clustering, reduce NF-κB activation	Attenuated neuroinflammation, reduced pro-inflammatory activation (59, 76)	Preclinical	Novel mechanism	Specificity concerns	Acute
Immune checkpoint	VISTA	VISTA agonists (Ab/Fc)	Upregulate ACOD1/itaconate, suppress glycolysis via TRIM28-HK2	Reduced infarction, improved neurological scores (29, 78)	Preclinical	Immunometabolic integration	Biologic delivery challenges	Acute (up to 6h)

2-DG, 2-deoxy-D-glucose; 4-OI, 4-octyl itaconate; ACOD1, aconitate decarboxylase 1; CPT1, carnitine palmitoyltransferase 1; FAO, fatty acid β-oxidation; HK2, hexokinase 2; IL-1β, interleukin-1β; NAD^+^, nicotinamide adenine dinucleotide; NAMPT, nicotinamide phosphoribosyltransferase; NF-κB, nuclear factor kappa-light-chain-enhancer of activated B cells; NLRP3, NLR family pyrin domain containing 3; NMN, nicotinamide mononucleotide; NR, nicotinamide riboside; Nrf2, nuclear factor erythroid 2-related factor 2; PFK1, phosphofructokinase 1; PFKFB3, 6-phosphofructo-2-kinase/fructose-2,6-biphosphatase 3; PHLDA1, pleckstrin homology-like domain family A member 1; PPAR, peroxisome proliferator-activated receptor; SCENITH: Single Cell ENergetIc metabolism by profiling Translation inhibition; SDH, succinate dehydrogenase; SIRT1, sirtuin 1; SphK1, sphingosine kinase 1; TLR4, toll-like receptor 4; TRIM28, tripartite motif-containing 28; VISTA, V-domain Ig suppressor of T cell activation.

### 4.5 Intervention timing and therapeutic windows

- Acute phase (0–72 h): Glycolysis inhibition (2-DG at 50–100 mg/kg i.p. at reperfusion, or 100 mg/kg daily for 7 days prior to ischemia in preconditioning protocols; Shikonin at 10–20 mg/kg i.p. at reperfusion) and itaconate derivatives (4-OI at 25–50 mg/kg i.p. immediately post-reperfusion) (29, 43, 70).
- Subacute phase (3–14 d): FAO promotion (PPAR agonists) and NAD^+^ precursor supplementation.
- Chronic phase (>14 d): Metabolic homeostasis maintenance.

These temporal stratifications are informed by preclinical studies that have explored pathway-specific interventions in cerebral ischemia and related models (29, 43, 78), though exact time windows may vary across experimental paradigms.

## 5 Current challenges and future perspectives

### 5.1 Decoding spatiotemporal metabolic heterogeneity

**Figure 4:**
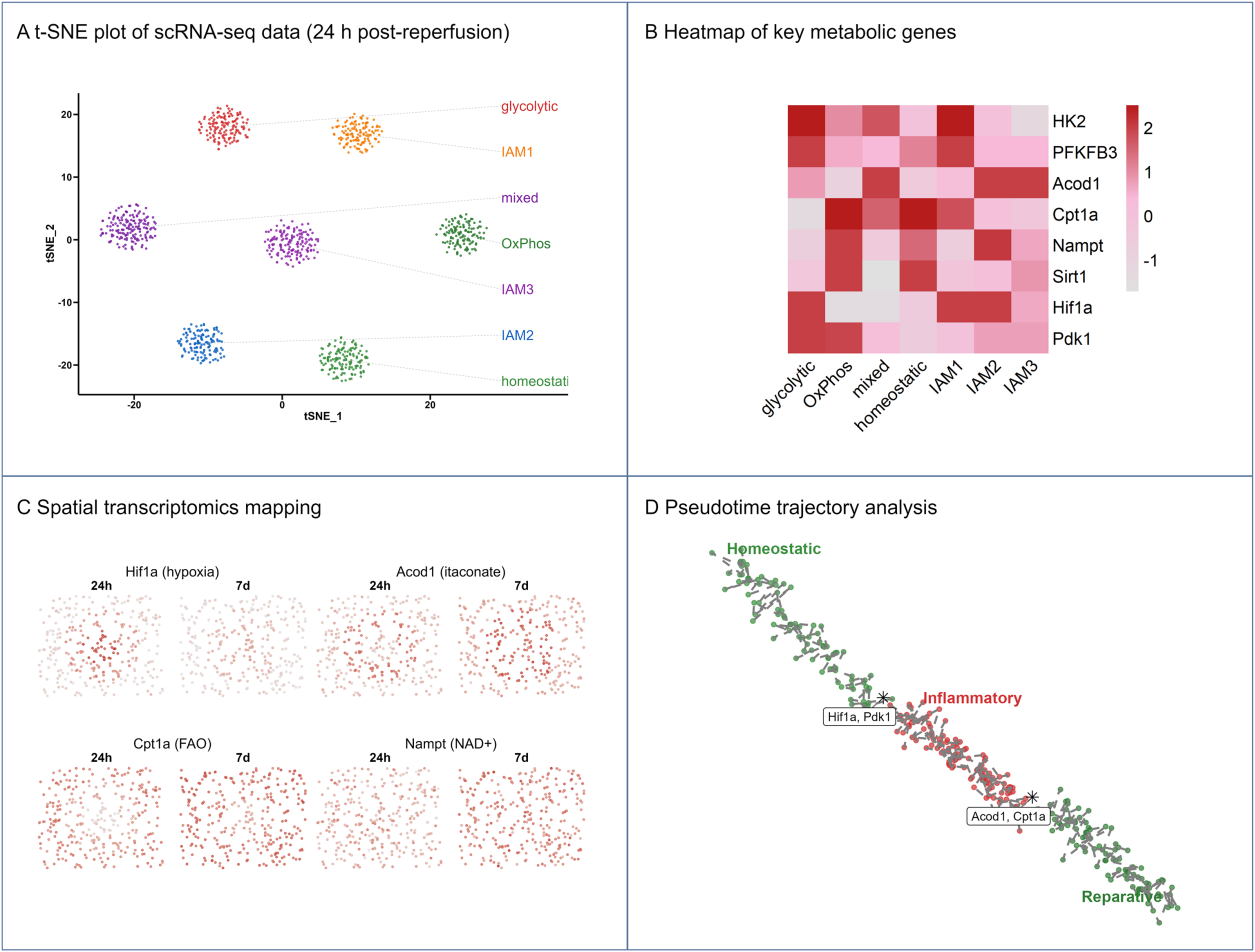
Spatiotemporal metabolic heterogeneity of microglia in CIRI. Single-cell and spatial profiling reveals distinct microglial metabolic states across different brain regions and time points following cerebral ischemia-reperfusion injury. **(A)** t-SNE plot of single-cell RNA sequencing data showing seven transcriptionally distinct microglial subclusters isolated from ischemic brain at 24 h post-reperfusion. Clusters are color-coded by predominant metabolic signature: glycolytic (red), oxidative phosphorylation (blue), mixed (purple), and homeostatic (green). **(B)** Heatmap displaying relative expression levels of key metabolic genes across identified microglial subpopulations. Expression levels are represented by a color gradient from gray (low) to dark red (high). **(C)** Spatial transcriptomics mapping of metabolic gene signatures in coronal brain sections at 24 h and 7 days post-reperfusion, showing regional heterogeneity between ischemic core (red), penumbra (orange), and distal regions (green). Representative genes (*Hif1a*, *Acod1*, *Cpt1a*, *Nampt*) illustrate distinct spatiotemporal expression patterns. **(D)** Pseudotime trajectory analysis illustrating the dynamic transition of microglial metabolic states from homeostatic (green) to inflammatory (red) to reparative (blue) phenotypes, with key metabolic checkpoint genes (*Hif1a*, *Pdk1*; *Acod1*, *Cpt1a*) indicated at branch points. (Empirical data adapted from 13; contrasts with conceptual timeline in **Figure 2**). Core, ischemic core; HM, homeostatic microglia; IAM1-3, ischemia-associated microglia subtypes; Pen, penumbra; RM, reparative microglia. Data are adapted from references (13) and presented as a schematic overview.

Single-cell RNA sequencing has identified seven transcriptionally distinct microglial subclusters following cerebral ischemia, exhibiting metabolic heterogeneity (13). Key ischemia-associated genes (Cd14, Csf1, Tlr2) have been subsequently validated in independent datasets (17). For a broader context on microglial biology, readers are referred to Prinz et al. (14). **Figure 2** provides a conceptual schematic of the temporal phases of microglial activation, serving as a framework for understanding the dynamic trajectory. In contrast, **Figure 4** presents empirical data-derived findings from recent single-cell and spatial omics studies (13), demonstrating actual transcriptional subclusters and their metabolic signatures. Pseudotime analysis revealed transitions from homeostatic to pathological states, highlighting potential therapeutic windows (**Figure 4**). Spatial metabolomics has now begun to map metabolite distributions across ischemic regions, revealing region-specific metabolic reprogramming in the ischemic core, penumbra, and even remote ipsilateral cortex (92, 93). For instance, genes such as *Hif1a*, *Acod1*, *Cpt1a*, and *Nampt* exhibit distinct spatiotemporal expression patterns across these regions (**Figure 4C**). Future research must leverage such technologies alongside single-cell metabolic flux analysis (e.g., SCENITH (94), a method with potential future applications in stroke metabolic profiling).

Despite these technological advances, significant limitations remain in determining actual metabolic flux in microglia within the living, post-ischemic brain. Most single-cell studies rely on transcriptomic proxies or static metabolite snapshots rather than direct flux measurements. *In vitro* extracellular flux analyses (e.g., Seahorse assays) provide high-resolution real-time metabolic parameters but cannot recapitulate the complex, dynamic microenvironment of the ischemic brain—including fluctuating oxygen tension, substrate availability, cell-cell interactions, and temporal changes in DAMPs and cytokines. Conversely, *in vivo* metabolic imaging techniques (e.g., hyperpolarized ¹³C magnetic resonance spectroscopy) offer real-time flux data but lack the cellular resolution needed to distinguish microglial metabolism from that of neurons, astrocytes, and infiltrating immune cells. Bridging this gap will require innovative approaches such as cell-type-specific metabolic labeling coupled with *in vivo* imaging or single-cell metabolomics platforms that capture flux dynamics rather than steady-state abundances.

### 5.2 Achieving cell-specific targeting

Nanotechnology-based carriers: Nanoparticles functionalized with ligands for microglial surface receptors (e.g., TREM2, CX3CR1, P2Y12) represent an emerging strategy for selective targeting, with recent advances in microglial biology (95) and nanomedicine (96) paving the way. Hyaluronic acid-based nanoparticles targeting CD44 (96) and Apoferritin nanocages (97) have been developed to deliver metformin, demonstrating enhanced efficacy in CIRI models through improved mitochondrial function and NLRP3 inhibition.

Microglia-promoter-driven gene therapies: Adeno-associated virus (AAV) vectors incorporating microglia-specific promoters (*CD68*, *CX3CR1*, *TMEM119*) enable selective transgene expression (98).

Prodrug strategies: Designing prodrugs requiring microglia-specific enzymatic activation could achieve cell-selective effects (72).

### 5.3 Bridging the translational gap

Validation in higher-order species and inclusion of aged animals, both sexes, and comorbidity models are critical (99, 100). Rigorous pharmacokinetic/pharmacodynamic (PK/PD) studies and biomarker development (cerebrospinal fluid/plasma itaconate, succinate, lactate; NAD^+^/NADH ratios; translocator protein-positron emission tomography (TSPO-PET) imaging for microglial activation) are essential (101, 102).

### 5.4 Integrating systems-level and sex-specific understanding

Gut-Brain Axis: Stroke induces gut dysbiosis and reduces short-chain fatty acid (SCFA) production, negatively correlating with outcomes (91). Butyrate supplementation produces sex-specific microglial responses (21, 103). Sex Differences: Females exhibit greater microglial ramification and different cytokine responses; sex hormones modulate microglial metabolism (104). Multi-Omics Integration is required to construct comprehensive “metabolome-inflammasome” networks (105).

### 5.5 Future directions: toward precision immunometabolic therapy

Building upon the challenges outlined above, future research should pursue the following strategic directions:

1. Mapping spatiotemporal heterogeneity (addressing 4.1): Construct single-cell metabolic atlases integrating spatial transcriptomics and metabolomics to identify novel microglial subpopulations and their dynamic metabolic signatures across CIRI progression.
2. Developing cell-specific delivery systems (addressing 4.2): Advance nanotechnology platforms, microglia-specific AAV serotypes, and prodrug strategies to achieve selective targeting while minimizing off-target effects.
3. Enhancing translational rigor (addressing 4.3): Implement multi-species validation, incorporate both sexes and aged/comorbid models, and establish validated biomarkers (metabolites, imaging) for patient stratification and treatment monitoring.
4. Integrating systems-level modifiers (addressing 4.4): Elucidate gut-brain axis contributions and sex-specific metabolic regulation to enable personalized therapeutic approaches.
5. Designing stage-specific combination regimens (integrating all challenges): Develop multi-stage treatment protocols combining complementary metabolic interventions aligned with the dynamic immunometabolic landscape, supported by rational combination therapies targeting synergistic nodes.

## 6 Conclusion

Microglial metabolic reprogramming is a dynamic and integral component of the neuroinflammatory response to CIRI, acting as a decisive “metabolic switch” controlling the balance between detrimental pro-inflammatory and protective reparative phenotypes. The four major metabolic pathways form an integrated network with extensive crosstalk (**Figure 3**). Targeted manipulation through metabolic enzyme inhibitors/activators, immunometabolic checkpoint regulators, metabolite supplementation, or multi-target natural products holds substantial neuroprotective potential (**Table 1**). Agents in active clinical development—including PPAR agonists (fenofibrate) and NAD^+^ precursors (NMN/NR)—offer the most immediate translational opportunities. Overcoming challenges in spatiotemporal heterogeneity characterization, cell-specific delivery, and biomarker development will accelerate the evolution of immunometabolic targeting from compelling concept to transformative therapy for CIRI, thereby providing a comprehensive conceptual framework and translational roadmap for precision immunometabolic intervention in ischemic stroke.
